# Liver-Directed Treatment Options Following Liver Tumor Recurrence: A Review of the Literature

**DOI:** 10.3389/fonc.2022.832405

**Published:** 2022-01-31

**Authors:** Christopher T. Aquina, Mariam F. Eskander, Timothy M. Pawlik

**Affiliations:** ^1^ Division of Surgical Oncology, Department of Surgery, The Ohio State University Wexner Medical Center, Columbus, OH, United States; ^2^ Digestive Health and Surgery Institute, AdventHealth Orlando, Orlando, FL, United States; ^3^ Division of Surgical Oncology, Department of Surgery, Robert Wood Johnson Medical School and Rutgers Cancer Institute of New Jersey, New Brunswick, NJ, United States

**Keywords:** recurrent liver tumors, hepatectomy, tumor ablation, liver transplant, transarterial chemoembolization (TACE), radioembolization of liver malignancies, SBRT (stereotactic body radiation therapy), recurrent liver tumors

## Abstract

Recurrence following curative-intent hepatectomy for colorectal cancer liver metastasis, hepatocellular carcinoma, or cholangiocarcinoma is unfortunately common with a reported incidence as high as 75%. Various treatment modalities can improve survival following disease recurrence. A review of the literature was performed using PubMed. In addition to systemic therapy, liver-directed treatment options for recurrent liver disease include repeat hepatectomy, salvage liver transplantation, radiofrequency or microwave ablation, intra-arterial therapy, and stereotactic body radiation therapy. Repeat resection can be consider for patients with limited recurrent disease that meets resection criteria, as this therapeutic approach can provide a survival benefit and is potentially curative in a subset of patients. Salvage liver transplantation for recurrent hepatocellular carcinoma is another option, which has been associated with a 5-year survival of 50%. Salvage transplantation may be an option in particular for patients who are not candidates for resection due to underlying liver dysfunction but meet criteria for transplantation. Ablation is another modality to treat patients who recur with smaller tumors and are not surgical candidates due to comorbidity, liver dysfunction, or tumor location. For patients with inoperable disease, transarterial chemoembolization, or radioembolization with Yttrium-90 are liver-directed intra-arterial therapy modalities with relatively low risks that can be utilized. Stereotactic body radiation therapy is another palliative treatment option that can provide a response and local tumor control for smaller tumors.

## Introduction

Colorectal cancer is the third most common cancer worldwide with the liver being the most common site of metastatic disease and primary liver cancer is the sixth most common cancer worldwide making the liver a very common site of disease (1). In fact, an estimated 149,500 patients in the United States will be diagnosed with colorectal cancer in 2021 among whom 25% will present with synchronous liver metastasis and 50% will eventually develop metachronous liver metastasis; in addition, an estimated 42,230 patients in the United States will be diagnosed with liver or intrahepatic bile duct malignancy in 2021 (2–4). Among these patients, approximately 10-20% of individuals with colorectal cancer liver metastasis (CRCLM), 20% of patients with hepatocellular carcinoma (HCC), and 50% of those with intrahepatic cholangiocarcinoma will undergo resection with curative intent (5–7). Unfortunately, recurrence rates are as high as 70% for each of these cancer types (8–10). We herein review currently available therapeutic modalities and outcomes associated with liver-directed treatment of patients with hepatic tumor recurrence.

## Methods

MEDLINE, PubMed and Web of Science databases were queried for published articles through August 31^st^, 2021 using the search terms *recurrence* AND {[(*colon*, *rectal*, *colorectal)* AND *(cancer, neoplasm, adenocarcinoma)* AND *liver metastasis*] OR *hepatocellular carcinoma* OR *cholangiocarcinoma*}. The results were reviewed to identify English language primary studies that investigated outcomes following re-intervention for liver recurrence following prior resection of colorectal cancer liver metastasis, hepatocellular carcinoma, or cholangiocarcinoma. Specific re-interventions included repeat hepatectomy, liver transplantation, radiofrequency (RFA) or microwave ablation (MWA), hepatic artery infusion chemotherapy, transarterial embolization, chemoembolization, radioembolization, and stereotactic body radiation therapy (SBRT). References embedded in publications were also reviewed to identify additional studies.

## Treatment of Recurrent Liver Tumors

### Colorectal Cancer Liver Metastasis (CRLM)

While 10-year overall survival can be as high as 20-30% following hepatectomy for colorectal cancer, up to 70% of patients will develop disease recurrence (8, 11). Factors associated with recurrence include a node-positive primary tumor, >3 liver metastases, and a liver metastatic lesion >4 cm (12). Among patients who develop recurrent disease following initial curative-intent treatment of CRLM, an estimated 43% develop liver-only recurrence, 35% have extrahepatic only disease, and 21% experience both intra- and extrahepatic recurrence (13). Among patients with suspected recurrence, the work-up should include clinical examination, liver and renal functional tests, serum carcinoembryonic antigen (CEA) level, and imaging consisting of computed tomography (CT) or magnetic resonance imaging (MRI) of the abdomen and pelvis (14, 15). Additional evaluation of the chest with CT scan is also mandatory.

Individuals with liver-only recurrence may be candidates for curative-intent resection and/or ablation. For patients who also have evidence of extrahepatic disease recurrence, systemic therapy +/- liver-directed therapy is warranted. Current first-line systemic therapy options include 5-fluorouracil or capecitabine in combination with oxaliplatin (FOLFOX or CAPEOX), irinotecan (FOLFIRI), or both agents (FOLFOXIRI) with the potential addition of a targeted agent (e.g., bevacizumab for *RAS* mutated tumors; cetuximab or panitumumab for *RAS* wild-type tumors). Among patients with deficient DNA mismatch repair and microsatellite instability (dMMR/MSI-H), the addition of checkpoint inhibitors (pembrolizumab, nivolumab, or ipilimumab) may be warranted (14, 15). In addition to systemic therapy, liver-directed treatment modalities include hepatic artery infusion pump therapy, arterial-directed embolic therapy, and SBRT.

Careful assessment of liver functional reserve prior to any liver-directed therapy is essential. For patients being considered for repeat hepatectomy, assessment for an adequate future liver remnant using CT or MRI volumetry is required to limit the risk of postoperative liver failure (16). In general, the future liver remnant (FLR) is adequate when the FLR is ≥ 20% of the total liver volume (TLV) in patients with a normal liver, ≥ 30% in patients who received chemotherapy, and ≥ 40% in those with hepatic fibrosis or cirrhosis (17). For patients in which there is a concern for an inadequate future liver remnant, preoperative portal vein embolization may be an option to induce hypertrophy of the anticipated future liver remnant (18). Another option is associating liver partition and portal vein ligation for staged hepatectomy (ALPPS). However, the reported mortality rate after ALPPS and resection of CRCLM was 9% raising concern about its safety (19). For patients with cirrhosis or suspected liver dysfunction, a history of gastrointestinal hemorrhage, ascites, encephalopathy, or thrombocytopenia with platelet count < 150,000/μL will impact risk of hepatectomy. The Child-Turcotte-Pugh Classification, which is calculated based on the degree of encephalopathy and ascites and measurements of serum bilirubin, serum albumin, and prothrombin time or international normalized ratio, should be estimated as resection is usually reserved for Class A and carefully selected Class B patients with cirrhosis due to increased risk of postoperative morbidity and mortality. Furthermore, portal hypertension should be assessed through endoscopy to evaluate for esophageal varices, as well as imaging to assess for ascites, splenomegaly, liver nodularity, portosystemic collaterals, and mesenteric vein thrombosis. If the diagnosis of portal hypertension is still in question following these less invasive diagnostic modalities, percutaneous or transjugular liver biopsy or hepatic venous pressure gradient (HVPG) measurement can be performed. A HVPG ≥ 6 mmHg indicates portal hypertension and a HVPG ≥ 10 mmHg has been associated with clinically significant portal hypertension and increased post-operative risk.

#### Repeat Hepatectomy

Repeat resection has been demonstrated to be a safe and effective treatment for recurrent CRLM. While reoperation can be challenging due to abdominal and perihepatic adhesions and altered hepatobiliary anatomy, postoperative 30-day mortality is less than 2%, and there is no difference in morbidity and mortality compared with initial liver resection for CRLM (20–22). In addition, a laparoscopic approach for repeat hepatectomy appears safe and may provide an advantage over open resection with one meta-analysis demonstrating lower intraoperative blood loss, less overall and major postoperative complications, shorter hospital stay, a higher R0 resection rate, and equivalent operative time, transfusion rate, and mortality (23). While there are no randomized controlled trials comparing resection to other treatment modalities for recurrent disease, hepatectomy remains the gold standard treatment modality for resectable recurrent CRLM.

In carefully selected patients, long-term survival with potential cure can be achieved with resection of recurrent liver disease. Across a multitude of single-institution retrospective studies, the reported 5-year overall survival following repeat hepatectomy for liver-only recurrence ranges from 27% to 67% (**Table 1**) (24–37). However, survival appears to be shorter after each subsequent liver resection for recurrence. In a study by de Jong et al. that included 246 patients with CRLM who underwent curative intent surgery with resection alone, RFA alone, or a combination of resection and RFA, 5-year overall survival was 47%, 33%, and 24% following the first, second, and third curative intent operations, respectively (21). As with any patient under consideration for a hepatectomy, preoperative assessment of patient performance status and adequate remnant liver volume is essential.

**Table 1:** Survival after repeat resection for colorectal cancer liver metastasis recurrence in the liver.

Authors	Years	Type	N	Median OS (months)	1-year OS	3-year OS	5-year OS
Adam et al. (24)	1984-2000	Single center	199	–	88%	54%	35%
Ahmad et al. (25)	1997-2003	Single center	19	48	95%	79%	44%
Battula et al. (26)	1998-2011	Single center	53	45	85%	61%	52%
Ishiguro et al. (27)	1985-2004	Single center	111	43	91%	74%	41%
Maeda et al. (28)	2000-2016	Single center	17	>60	–	–	52%
Nanji et al. (29)	2002-2009	Multicenter	78	45	–	–	45%
Park et al. (30)	2003-2016	Single center	70	62	–	62%	50%
Sa Cunha et al. (31)	1985-2000	Single center	40	32	–	55%	31%
Saiura et al. (32)	1999-2008	Single center	73	NR	–	75%	67%
Shaw et al. (33)	1987-2005	Single center	66	56	94%	68%	44%
Thelen et al. (34)	1988-2006	Single center	94	–	89%	55%	38%
von Heesen et al. (35)	2001-2006	Single center	23	–	–	66%	27%
Yamamoto et al. (36)	1985-1997	Single center	75	30	–	48%	31%
Yamazaki et al. (37)	2004-2011	Single center	37	–	92%	52%	36%

Includes studies with ≥10 patients who underwent repeat resection.

OS, overall survival following second hepatectomy; NR, not reached.

Prognostic factors associated with increased risk of recurrence or worse survival following repeat hepatectomy are a history of synchronous liver metastasis, an initial CRLM ≥5 cm, positive surgical margins at initial resection, a relapse-free interval of less than one year, the presence of multiple liver lesions or extrahepatic disease at the time of second hepatectomy, and positive surgical margins at repeat hepatectomy (27, 36, 38, 39). Limited evidence is available regarding the benefit of perioperative systemic therapy in patients who undergo repeat resection. However, the European Organization for Research and Treatment of Cancer (EORTC) intergroup trial 40983 demonstrated a progression-free survival benefit, but there was no overall survival benefit among patients who received perioperative FOLFOX4 compared with surgery alone for resectable CRLM (40).

#### Tumor Ablation

Tumor ablation in the form of RFA or MWA is a locoregional treatment that can be utilized alone or in combination with resection with curative or palliative intent. It appears to be an effective and safe treatment modality in patients with CRLM ≤ 3 cm in diameter who are otherwise not good candidates for resection due to unfavorable tumor location, comorbidity burden, or functional status (41). While there are no randomized controlled trials that have assessed differences in outcomes between ablation and repeat hepatectomy, there are several recent retrospective studies available comparing short-term morbidity and survival between the two treatment modalities.

In a single-institution retrospective study of 64 consecutive patients in the United Kingdom who developed liver-limited recurrence following hepatectomy between 2010 and 2015, 33 patients were treated with RFA or MWA, and 31 patients were treated with repeat hepatectomy (42). Overall morbidity (12.1% vs 38.7%, p=0.02) and length of stay (1 vs 5 days, p<0.001) were significantly lower in the ablation group. After a median follow-up of 36.2 months across the study cohort, median overall survival was the same between the two groups at 33.3 months (p=0.45). However, median progression-free survival was longer (10.2 vs 4.3 months, p=0.002) among patients in the repeat resection group. Given the lower morbidity rate, yet shorter progression-free survival associated with ablation, the authors concluded that the choice between ablation and resection should be made on a personalized basis. In another single-institution retrospective study in China that included 194 patients with recurrent CRLM, 50 patients underwent repeat liver resection, and 144 patients underwent RFA (43). Indications for RFA included ≤3 tumors with a maximum diameter of ≤5 cm or >4 tumors with a maximum diameter of ≤3 cm. In propensity-matched analyses, there was no significant difference in complication rates, disease-free survival, or overall survival. However, postoperative length of stay (14.5 vs 10.6 days, p=0.006) was longer in the hepatic resection group. Similar results were observed in a retrospective study utilizing the Amsterdam Colorectal Liver Met Registry. Among 136 patients, 100 individuals were treated with repeat thermal ablation and 36 underwent repeat liver resection between May 2002 and December 2020. There were no significant difference in overall survival, distant progression-free survival, local tumor progression-free survival, or complications between the two groups. However, mean length of stay was significantly shorter among patients in the thermal ablation group (2.1 vs 4.8 days, p=0.009). While these retrospective studies suggest that repeat ablation for recurrent CRCLM ≤ 3 cm in diameter is equivalent to repeat hepatectomy with respect to survival and appears to be associated with lower morbidity, the awaited phase III randomized controlled COLLISION trial comparing 5-year survival between these two groups should provide more definitive evidence (44).

#### Hepatic Arterial Infusion Pump Chemotherapy

Hepatic arterial infusion pump (HAIP) chemotherapy with floxuridine has been shown to have CRLM response rates as high as 92%, conversion-to-resection rates as high as 47% for initially unresectable CRLM, and improvement in 2-year hepatic disease-free survival from 60% to 90% in clinical trials (45–47). Therefore, HAIP chemotherapy may be an effective treatment modality for patients with initially unresectable recurrent CRLM or as adjuvant therapy to reduce the risk of liver re-recurrence by treating residual liver micrometastases. In the only study to investigate outcomes following HAIP chemotherapy for recurrent CRCLM, Buisman et al. performed a retrospective cohort study of 374 patients with liver-only disease who underwent ablation and/or resection of recurrent CRLM (48). A total of 81 patients were treated with HAIP chemotherapy for a maximum of 6 cycles; patients who received HAIP chemotherapy were significantly more likely to be < 70 years old at the time of the index CRLM, to be female, to have had positive primary tumor nodal status, and to have received perioperative systemic chemotherapy (p<0.05). Despite differences between the two groups, adjuvant HAIP chemotherapy was independently associated with improved hepatic disease-free survival (hazard ratio [HR]=0.60, 95% confidence interval [CI]=0.38-0.93, p=0.02) and overall survival (HR=0.59, 95% CI=0.38-0.92, p=0.02) compared with patients who did not receive adjuvant HAIP chemotherapy. While these results are promising, there are currently no randomized controlled trial data with respect to HAIP chemotherapy for recurrent CRCLM.

#### Arterially Directed Embolic Therapy

Transarterial chemoembolization (TACE) is a minimally invasive treatment option for patients with unresectable CRCLM that is relatively well-tolerated and may provide a survival benefit, as well as facilitate conversion to resectability through local delivery of high-dose chemotherapy followed by arterial occlusion. While no study investigating its oncologic benefit for recurrent CRLM was identified, two small randomized trials have investigated the effect of TACE with drug-eluting beads preloaded with irinotecan (DEBIRI) on initial CRLM (49, 50). In a multi-institutional phase III trial by Fiorentini et al, 74 patients with unresectable CRLM who no radiological evidence of extrahepatic disease were randomized to receive DEBIRI or systemic chemotherapy with FOLFIRI; the DEBIRI group had longer median overall survival (22 vs 15 months, p=0.03), longer progression-free survival (7 vs 4 months, p=0.006), and longer median duration of improved quality of life (8 vs 3 months, p=0.00002) (49). In another multi-institutional phase III trial by Martin et al, 60 patients who were chemotherapy-naïve with liver dominant disease defined as ≥80% of the tumor burden confined to the liver were randomized to modified FOLFOX, bevacizumab, and DEBIRI (mFOLFOX-DEBIRI) or systemic therapy alone with modified FOLFOX and bevacizumab (mFOLFOX) (50). The mFOLFOX-DEBIRI group had a higher overall response rate at 2 months (78% vs 54%, p=0.02) and a higher rate of conversion to resectability (35% vs 16%) compared with mFOLFOX alone.

Another local treatment option for unresectable CRLM refractory to systemic chemotherapy is selective internal radiotherapy in which yttrium-90 (^90^Y)-tagged glass or resin microspheres are selectively delivered to the tumor *via* the hepatic artery and provide high doses of radiation. ^90^Y is typically well-tolerated with fatigue, nausea, and vomiting being the most common side effects (51). While there are limited data regarding its efficacy for patients who underwent prior resection of CRLM, one large multicenter observational study by Hickey at al included 531 patients who underwent ^90^Y radioembolization of CRLM of which 98 patients had undergone prior resection (52). Median overall survival for the entire study cohort from the time of first ^90^Y treatment was 10.6 months with a longer survival benefit observed among individuals without extrahepatic disease (14.4 vs 6.6 months, p<0.001). ^90^Y radioembolization may also allow for conversion to resection with a conversion rate as high as 29% in one small study that included 14 patients with CRCLM (53).

#### Stereotactic Body Radiation Therapy (SBRT)

SBRT allows for higher doses of radiation over fewer treatment fractions and has demonstrated good local control of CRLM with low toxicity in small studies (54). In a single institution retrospective observational study by Viganò et al. that included 206 consecutive patients with recurrent CRLM who had undergone liver resection between 2004 and 2013, local disease control and overall survival were compared among patients who underwent repeat resection, RFA, or SBRT (55). Among 14 patients who underwent SBRT, the 2-year local disease control rate was 70.8% versus 90.9% for repeat liver resection (p=0.051) and 56.4% for RFA (p=0.536). While larger prospective studies are needed, SBRT can be considered in patients with unresectable liver-limited disease at centers with expertise.

#### Salvage Liver Transplantation (SLT)

Liver transplantation for unresectable CRCLM remains controversial due to the risk of high recurrence with a 1-year disease-free survival rate of only 39% (56). However, in areas where organ scarcity is not an issue, it may provide an overall survival benefit versus systemic therapy alone (estimated 5-year overall survival only 10%) (57). In the SECA-1 trial that included 21 patients who underwent liver transplantation for unresectable CRCLM, time from primary tumor resection to liver transplantation > 2 years, treatment response or stable disease following chemotherapy, pre-transplantation serum CEA < 80 μg/L, and tumor diameter < 5.5 cm were associated with more favorable survival outcomes (58). In the SECA-1 (N=4) and subsequent SECA-2 (N=4) trials, only 8 patients with recurrent CRCLM following prior liver resection were included in the studies. Furthermore, stratified outcome data by recurrence status were not reported (58, 59). Given the overall paucity of data regarding liver transplantation for CRCLM and its association with high recurrence rates in the small studies that have been published, SLT should only be performed for recurrent CRCLM as part of a comprehensive transplant program after extensive multi-disciplinary review.

### Hepatocellular Carcinoma (HCC)

For patients without cirrhosis and significant comorbidity burden, hepatectomy is often the treatment modality of choice for resectable HCC. In contrast, for patients with cirrhosis meeting the Milan criteria of a solitary tumor ≤ 5 cm in diameter or no more than three tumor nodules all measuring ≤ 3 cm in diameter without major vessel or extrahepatic involvement, liver transplantation is the treatment of choice (60). Recurrence rates have been reported to be as high as 70% following hepatectomy and 18% following liver transplantation, which are most often detected on surveillance multiphasic CT or MRI of the liver (9, 61). Following liver resection, most recurrences occur early with approximately 75% occurring within 2 years, and approximately 90% of recurrences occurring in the remnant liver (62). Recurrence patterns following liver transplantation differ from that of initial partial hepatectomy. While most recurrences also occur early with a median time to recurrence of 12 months, 23% develop liver-only recurrence, 39% develop both intra- and extrahepatic recurrence, and 39% develop isolated extrahepatic recurrence (61). For patients with suspected recurrence, work-up should include clinical examination, liver and renal functional tests, serum alpha-fetoprotein (AFP) level, and imaging consisting of multiphasic liver protocol CT abdomen/pelvis or intravenous contrast-enhanced MRI abdomen/pelvis, CT chest, and bone scan in the setting of skeletal symptoms (63).

Patients with liver-only recurrence may be candidates for curative-intent repeat hepatectomy, liver transplantation, or ablation. For individuals with unresectable disease, other liver-directed therapy modalities include TACE, ^90^Y, and SBRT. For patients who also have evidence of extrahepatic disease recurrence, enrollment in a clinical trial or systemic therapy with atezolizumab + bevacizumab or a tyrosine kinase inhibitor such as sorafenib or lenvatinib may be warranted (63).

Given the high prevalence of cirrhosis in patients with HCC, a full assessment of underlying liver function and portal hypertension prior to any intervention is especially important. While there are no published algorithms regarding liver function, tumor stage, and first-line treatment recommendations for recurrent HCC, the Barcelona Clinic Liver Cancer and Japan Society of Hepatology treatment strategy algorithms can be extrapolated and utilized as treatment guides (64, 65). In general, resection is an option only in patients with Child-Pugh Class A cirrhosis or highly selected patients with Child-Pugh B cirrhosis without evidence of portal hypertension who have a FLR : TLV ratio ≥ 40% on CT or MRI volumetry. Liver transplantation should be considered in patients with an inadequate FLR who would not be a candidate for repeat hepatectomy. Ablation can be considered in patients with portal hypertension who have small (<3-5 cm) lesions. Hepatectomy, ablation, TACE, ^90^Y, SBRT, and systemic therapy are generally contraindicated in patients with Child-Pugh Class C cirrhosis; salvage liver transplantation may be the only option in those individuals who meet transplant criteria and have acceptable functional status.

#### Repeat Hepatectomy

Repeat hepatectomy for recurrent HCC is safe with comparable complication rates to initial hepatectomy and is often the curative-intent treatment of choice (66). While patients who develop recurrent HCC following liver transplantation have a worse prognosis versus patients who undergo partial hepatectomy with a median overall survival after recurrence of 10-13 months versus 24 months, hepatectomy is also the treatment of choice in post-transplant patients (67). As most patients with HCC have underlying liver disease, assessment of preoperative liver function, identification of portal hypertension, and determination of an adequate functional liver remnant are key in appropriate patient selection to limit the risk of postoperative morbidity. In addition, for patients who underwent initial partial hepatectomy, consideration should be given to the time interval from initial resection to recurrence. Early recurrences within one year appear to arise from intrahepatic metastasis from the primary tumor and are associated with a poor prognosis; in contrast, recurrences beyond one year are more likely to be multicentric occurrences arising in the setting of chronic liver disease that are associated with a better prognosis (68–70). Given that early recurrence is typically associated with unfavorable tumor biology including microvascular invasion, satellite micrometastases, and lower response rates to potentially-curative treatment such as repeat hepatectomy, tumor ablation, and salvage liver transplantation (SLT), initial treatment with less morbid therapy, such as TACE or combined therapy with TACE and RFA or a tyrosine kinase inhibitor, should be considered in these patients (68, 71).

Retrospective studies have demonstrated that partial hepatectomy can lead to long-term survival in appropriately selected patients following both initial hepatectomy and liver transplantation. Five-year overall survival range from 22% to 73% following repeat resection for recurrent HCC in the liver and 31% to 35% following metastasectomy for recurrent HCC following liver transplantation (**Table 2**) (66, 72–85). However, the main limitation of repeat resection is that only 15% to 30% of patients are eligible for repeat hepatectomy due to an inadequate future liver remnant, cirrhosis, multifocal disease, or gross vascular invasion (71).

**Table 2:** Survival after resection for hepatocellular carcinoma (HCC) recurrence in the liver.

Authors	Years	Type	N	Median OS (months)	1-year OS	3-year OS	5-year OS
^*^After initial partial hepatectomy for primary HCC							
Ho et al. (72)	2001-2007	Single center	54	–	–	–	72%
Huang et al. (73)	1995-2010	Single center	82	–	71%	41%	22%
Itamoto et al. (74)	1990-2004	Single center	84	–	88%	67%	50%
Li et al. (75)	1997-2015	Single center	103	65	92%	–	54%
Lu et al. (76)	2004-2015	Single center	138	–	92%	82%	73%
Midorikawa et al. (77)	2000-2017	Single center	273	68	–	–	58%
Minagawa et al. (78)	1994-2000	Single center	67	–	93%	70%	56%
Sun et al. (79)	1997-2003	Single center	57	–	70%	61%	31%
Wang et al. (80)	2004-2010	Single center	128	–	98%	84%	64%
Wu et al. (66)	1990-2007	Single center	149	–	–	–	56%
**^†^After initial liver transplantation for primary HCC**							
Bodzin et al. (81)	1984-2014	Single center	25	28	–	–	–
Fernandez-Sevilla et al. (82)	1991-2013	Single center	22	35	–	–	–
Huang et al. (83)	1997-2012	Single center	15	–	92%	51%	35%
Regalia et al. (84)	1987-1996	Multicenter	7	–	–	57%	–
^‡^Sapisochin et al. (85)	2000-2012	Multicenter	38	–	75%	60%	31%

^*^Includes studies with ≥50 patients who underwent repeat resection.

^†^Includes resection of extrahepatic metastases.

^‡^Includes resection and ablation.

OS, overall survival following hepatectomy for recurrent HCC.

In a systematic review of 22 observational studies, prognostic factors associated with increased risk of recurrence or worse survival following repeat hepatectomy were tumor size ≥ 3 cm, multifocal disease, micro- and macrovascular invasion, relapse-free interval of less than one year, cirrhosis, and receipt of a blood transfusion (86). Shorter time to recurrence was significant in 89% of the studies that reported time to recurrence. As mentioned, early recurrence is strongly associated with intrahepatic metastasis from the primary tumor and unfavorable tumor biology. Therefore, less aggressive initial therapy for recurrent HCC is warranted in most of these patients. For patients who underwent initial liver transplantation, unfavorable prognostic factors following metastasectomy for recurrent HCC include pretransplant model for end-stage liver disease (MELD) score > 23, elevated pretransplant neutrophil-to-lymphocyte ratio, microvascular invasion in the explanted liver, shorter time to recurrence, AFP level > 100 ng/mL, intrahepatic recurrence, and multifocal recurrence (81, 82, 85).

#### Salvage Liver Transplantation (SLT)

Since the first report of SLT for recurrent HCC by Majno et al. in 2000, subsequent studies have demonstrated the feasibility of liver transplantation for intrahepatic HCC recurrence following partial hepatectomy (87, 88). Five-year overall survival of 42% to 73% and 5-year recurrence-free survival rates of 32% to 81% following SLT for recurrent HCC have been reported across various retrospective and prospective studies (89–95). While there are no randomized controlled trials comparing repeat resection to SLT, numerous observational studies and several meta-analyses have been performed. For example, in a meta-analysis by Wang et al. that included 840 patients across 7 retrospective studies, SLT had improved 3-year (odds ratio [OR]=3.23, 95% CI=1.45-7.20, p=0.004) and 5-year disease-free survival (OR=4.79, 95% CI=1.88-12.25, p=0.001) compared with repeat hepatectomy; however, there was no difference in overall survival between the two groups (96). Similarly, in a meta-analysis by Kostakis et al. that included 516 patients across 3 prospective and 4 retrospective studies, SLT was associated with longer disease-free survival (HR=0.42, 95% CI=0.25-0.70, p=0.0009) compared with repeat liver resection; in addition, there was no difference in postoperative mortality or overall survival between the two groups (97). However, SLT was associated with worse short-term outcomes compared with repeat hepatectomy including higher blood loss, longer operative time, longer length of stay, and higher postoperative morbidity. In a different meta-analysis by Zheng et al. that included 2,818 patients from 21 retrospective studies, the authors compared overall and recurrence-free survival between SLT, repeat hepatectomy, RFA, TACE, and SBRT and ranked the modalities (98). With respect to overall survival, the ranking from most benefit to least benefit was SLT, repeat hepatectomy, SBRT, RFA, and TACE. SLT had significantly better recurrence-free survival compared with each of the treatment modalities. However, similar to the other meta-analyses, there was no significant difference in overall survival between repeat hepatectomy and SLT.

While there are not currently any official guidelines regarding SLT in the setting of recurrent HCC, various liver transplantation criteria, such as Milan or University of California San Francisco (UCSF) criteria, are typically applied by different institutions to identify candidates for SLT (60, 99). Given the scarcity of organs, repeat resection is preferred for patients with resectable intrahepatic disease and adequate liver function with SLT being reserved for patients who develop cirrhosis after hepatectomy for the primary HCC or who have unresectable disease but meet liver transplantation criteria. Ideally, SLT should be limited to patients with recurrent HCC who meet the stricter Milan criteria or carefully selected patients who meet the UCSF criteria, without microvascular invasion, and within 6 months of HCC recurrence (71).

#### Tumor Ablation

Compared with repeat hepatectomy and SLT, RFA and MWA are minimally invasive procedures with generally much lower morbidity, have lower rates of post-procedure liver dysfunction, and can be performed on a repeat basis (71, 100). Furthermore, in appropriately selected patients with smaller tumors, 5-year overall survival of 26% to 71% can be achieved (72, 76, 80, 83, 101–105). Ablation can also be utilized for bridging or downstaging to transplant (106). However, 5-year recurrence-free survival is lower with rates ranging from 0% to 30% across studies (80, 83, 101, 103, 104). While MWA has become the preferred modality at many institutions due to faster ablation times, less susceptibility to heat-sink effect, and the ability to perform multiple ablations simultaneously compared with RFA, MWA and RFA. These ablative modalities appear to have similar recurrence rates and overall survival in the treatment of primary HCC (107).

There is currently only one randomized controlled trial comparing repeat hepatectomy to RFA for recurrent HCC. Xia et al. randomized 240 patients with early-stage recurrent HCC defined as a solitary lesion ≤ 5 cm in diameter or ≤3 nodules each measuring ≤ 3 cm in diameter without evidence of macroscopic vascular invasion or extrahepatic metastases to either repeat hepatectomy or percutaneous RFA (108). There were no significant differences in outcomes between the repeat hepatectomy and RFA groups with respect to median overall survival (47.1 vs 37.5 months, p=0.17), 5-year overall survival (43.6% vs 38.5%, p=0.29), median recurrence-free survival (38.9 vs 25.8 months, p=0.09), and 5-year recurrence-free survival (36.2% vs 30.2%, p=0.09). However, in subgroup analysis of patients with a nodule > 3 cm in diameter, repeat hepatectomy was associated with improved overall survival (HR=0.55) and recurrence-free survival (HR=0.66) versus RFA. Therefore, repeat resection may be associated with better local disease control for larger tumors > 3 cm in diameter; RFA, however, had similar efficacy to repeat hepatectomy for smaller tumors measuring ≤ 3 cm in diameter. While these findings suggest that repeat hepatectomy and ablation may have equivalent efficacy for recurrent tumors ≤ 3 cm, additional studies comparing outcomes between repeat hepatectomy and ablation stratified by the number and size of recurrent tumors may better clarify the optimal therapeutic approach.

#### Arterially Directed Embolic Therapy

TACE is the most common treatment modality utilized for recurrent HCC following initial resection (71). However, there can be a significant risk of worsened liver dysfunction following the procedure among patients with underlying cirrhosis who have undergone prior hepatectomy. To help prevent Child-Pugh grade deterioration, the up-to-seven criteria, which is defined as the sum of the diameter of the largest tumor and the number of tumors, or the Mac-2 binding protein glycosylation isomer (M2BpGi) biomarker to assess liver fibrosis can be utilized to identify patients who are less likely to tolerate TACE (71, 109, 110). While TACE has been demonstrated to be inferior to repeat hepatectomy and SLT, it can improve survival among patients with early recurrence or multifocal disease with 5-year overall survival of 12% to 56% (66, 72, 77, 80, 98, 111–113). Similar to ablation, TACE can also be utilized for bridging or downstaging to transplant (106). While bland transarterial embolization (TAE) also has been demonstrated to be safe and effective for recurrent HCC, most studies have investigated the efficacy of TACE as arterially directed embolic therapy, and conventional TACE is the only transarterial modality found in randomized trials to provide a survival benefit compared with supportive care alone in patients with unresectable HCC not amenable to liver transplantation or ablation (114, 115). Therefore, TACE is generally the preferred treatment modality.

Selective internal radiotherapy with ^90^Y appears to be a safe alternative treatment option to TACE, especially in patients who are not candidates for TACE due to portal vein thrombosis (106, 116). It also appears to have similar efficacy to TACE (71). However, data are currently lacking with respect to outcomes following the use of ^90^Y for recurrent HCC following initial liver resection.

#### Stereotactic Body Radiation Therapy (SBRT)

SBRT can be utilized in patients with Child-Pugh Class A or early Class B cirrhosis with adequate liver volume outside of the radiation field. However, it should be avoided in patients with Child-Pugh score ≥ 8 due to increased risk of radiation-induced hepatic toxicity unless it is being utilized in the context of a clinical trial or as a bridge to SLT (117). While there are limited data regarding SBRT for recurrent intrahepatic HCC, a retrospective study by Shen et al. reported favorable outcomes following SBRT (118). Among 30 patients who underwent SBRT for recurrent HCC between 2008 and 2017, median overall survival was 50 months, the 1-year overall survival was 78% and 3-year overall survival was 58%. The authors performed a propensity score matching analysis comparing outcomes between 22 patients who underwent SBRT and 37 patients who underwent TACE and observed that SBRT was associated with improved 1-year overall survival (73% vs 38%, p=0.003) and 3-year overall survival (67% vs 0%, p < 0.001) versus TACE. There are also reports that SBRT can safely and effectively utilized as a bridge to transplantation for HCC (119, 120). In addition, repeat SBRT appears feasible with minimal toxicity in the setting of HCC recurrence following an initial course of SBRT (121). While these results are promising, prospective studies are needed.

### Cholangiocarcinoma

The liver is the most common site of recurrence after resection of intrahepatic cholangiocarcinoma (iCCA) (122). In a large German series of 202 patients with resected iCCA, 60.9% had a recurrence at a median of 7.5 months after resection, and 44% recurred in the liver only (123). In another Italian series of the 140 patients who underwent surgery for iCCA, 58.2% of the patients who recurred had liver metastasis (124). While hepatic recurrence is also common in hilar and distal cholangiocarcinoma, repeat resection is rarely possible (125). Therefore, most data on treatment of recurrent intrahepatic disease related to cholangiocarcinoma has focused on iCCA. Unfortunately, there is a paucity of prospective data to guide treatment decisions for intrahepatic recurrence.

Among patients with suspected intrahepatic recurrence, the work-up should include clinical examination, liver and renal functional tests, serum CEA level, serum CA 19-9 level, and imaging consisting of multiphasic CT or MRI of the chest, abdomen, and pelvis. Individuals with unifocal recurrence and no evidence of extrahepatic disease may be candidates for curative-intent resection and/or ablation. For patients who also have evidence of extrahepatic disease recurrence, systemic therapy is warranted. The currently preferred multi-agent regimen is gemcitabine and cisplatin, but other first-line options include 5-fluorouracil or capecitabine and a platinum-based agent consisting of oxaliplatin or cisplatin, gemcitabine and albumin-bound paclitaxel, capecitabine, or oxaliplatin, and single-agent therapy with 5-fluorouracil, capecitabine, or gemcitabine. Targeted therapy with entrectinib or larotrectinib can be considered in patients with NTRK gene fusion-positive tumors, and pembrolizumab can be considered in patients with MSI-H/dMMR tumors (63).

For patients being considered for liver-directed therapy, liver function should be assessed, and CT or MRI volumetry should be obtained for repeat hepatectomy candidates. For patients in which there is a concern for an inadequate future liver remnant, preoperative portal vein embolization should be considered. ALPPS should be avoided as it is associated with a mortality rate as high as 27% for cholangiocarcinoma (19).

#### Repeat Hepatectomy

Few studies, all retrospective, have examined survival outcomes following repeat liver resection, and the results have been overall favorable for highly selected patients. Patients who have a repeat resection survive longer than patients who do not undergo surgery (126). Compared with chemotherapy or other local therapies, surgery has been associated with improved survival outcomes (127). In a multicenter analysis, patients who underwent repeat liver resection had longer median survival (26.1 months) versus individuals who underwent ablation (25.5 months) or intra-arterial therapies (9.6 months) (p = 0.01) (10). Bartsch et al. reported no difference in survival among patients who were re-resected versus patients who had no recurrence at all (127). However, these studies must be cautiously interpreted due to small sample sizes and the potential for selection bias. As there is no randomized or prospective data, there are no official guidelines regarding repeat hepatectomy. The European Association for the Study of the Liver suggests either resection or ablation for “a small subset” of patients with liver-only recurrence (128). It appears reasonable to resect recurrent iCCA if an R0 resection of all hepatic lesions can be achieved and there is no evidence of extrahepatic disease. Surgical candidates should be carefully selected for favorable tumor biology, and some centers advocate for re-resection only after a disease-free interval of over 3 months (127). Other criteria include good functional status and an appropriately sized liver remnant. Palliative resections should not be performed (129).

Using these criteria, only about 10-25% of patients with recurrence are eligible for resection (10, 126, 130, 131). In one of the larger multi-institutional studies, 400 patients developed intrahepatic-only recurrences, and 190 (47.5%) underwent some form of liver-directed therapy. Among this cohort, only 28.5% had a hepatic resection (10). In a multi-center Japanese study, the re-resection rate was 31% for patients with intrahepatic-only recurrences occurring after a year from the initial operation. For patients with earlier recurrences, repeat surgery was performed in only 6.8% (132). **Table 3** presents survival data after re-resection of iCCA liver recurrences. The heterogeneity of these data can be attributed to the site of recurrence, extent of resection, timing of surgery, and the use of systemic therapy.

**Table 3:** Survival after repeat resection for intrahepatic cholangiocarcinoma recurrence in the liver.

Authors	Years	Type	N	Median OS (months)	1-year OS	3-year OS	5-year OS
Bartsch et al. (133)	2008-2017	Multicenter	113	65.2	98%	78%	57%
Hyder et al. (134)	1990-2011	Multicenter	33	25.8 RTDS	–	–	–
Kamphues, et al. (135)	2002-2008	Single center	13	51^*^	–	–	–
Konstadoulakis et al. (136)	1991-2005	Single center	7	20 RTDS	–	–	–
Langella, et al. (124)	2002-2020	Single center	21	31	–	–	–
Murakami et al. (137)	–	–	5	26 RTDS	–	–	–
Saiura et al. (138)	1995-2008	Single center	4	–	–	75%	50%
Si et al. (129)	2005-2013	Single center	72	45.1	97%	67%	412%
Souche et al. (130)	1997-2012	Multicenter	10	25 RTDS	–	40%	–
Spolverato et al. (10)	1990-2013	Multicenter	41	26.1^*^ RTDS	–	–	–
Sulpice et al. (126)	1997-2011	Single center	4	–	100%	100%	75%
Yoh et al. (139)	1993-2015	Single center	7	–	–	71%	–
Zhang et al. (140)	2007-2011	Single center	32	20.3 RTDS	84%	17%	–

OS, overall survival.

^*^Some patients also underwent ablation.

Other studies have highlighted the drawbacks of liver-directed therapy, with more than half of patients in one multi-institutional study having a second recurrence within a year of repeat resection (10). Several studies have attempted to understand the factors that influence the benefit of a repeat resection. In one study, tumor biology (disease-free interval of ≤12 months) and extent of initial liver resection (major hepatectomy) decreased the likelihood that repeat liver resection would results in a long-term benefit (124). Margin status, CA 19-9 at time of primary resection, and time to recurrence have been identified as other important predictors of long-term outcome (133). In a large study of 72 patients who underwent R0 repeat resection for recurrent iCCA, recurrent tumor size larger than 3 cm, multiple recurrent nodules, cirrhosis, and time to recurrence of less than one year were all negatively associated with time from recurrence to death (129).

To date, systemic chemotherapy has often been used and was often gemcitabine-based. Adjuvant therapy with capecitabine has generally become the standard of care after the BILCAP trial was published in 2019, and its impact on eligibility for and survival after repeat resection is not yet known (141).

#### Tumor Ablation

Ablation for recurrent iCCA can also provide good local control, provided that tumors are smaller than 5 cm and not located near large vessels or in a subcapsular location (142). In a retrospective Korean study, 20 patients with 29 recurrent lesions were identified (143). Tumors had to be 5 cm or smaller in diameter, with no more than three tumors, as well as no vascular invasion or extrahepatic metastasis. Ablation was performed percutaneously under either conscious sedation or local anesthesia. Technical success (complete ablation on imaging 1 month after the procedure) was achieved in 97% of tumors. Mean local tumor progression-free survival was 29.8 months and was significantly longer in patients with tumors under 1.5 cm. Overall survival at one year was 70% but decreased to 21% at 3 years. A small Chinese study used slightly different inclusion criteria: one lesion under 7 cm in diameter, up to three lesions under 3 cm each, tumors visible on ultrasound, no portal vein thrombosis, Child-Pugh grade A or B, platelet count above 50,000, and control of extrahepatic metastasis (144). Overall, 18 RFA treatments were performed in 12 patients with post-hepatectomy recurrence. Ablation effectiveness was 94.7%. However, during a median follow-up of 29.9 months after ablation, 5 patients needed repeat ablation due to recurrence. Median local recurrence-free survival was 21 months.

In some studies, using ablation to treat recurrent iCCA has yielded comparable survival to hepatic resection. In a retrospective Chinese study, 32 patients were treated with margin-negative repeat resection and 77 with RFA or MWA (140). The difference between indications for the two therapies were that there were no tumor size limitations for resection whereas tumors had to be under 5 cm for ablation. Median overall survival (20.3 vs. 21.3, p = 0.996) and disease-free survival (9.07 vs. 6.8 months, p = 0.692) were not significantly different between hepatic resection and thermal ablation. While two patients who underwent ablation developed liver abscesses, the overall rate of complications for thermal ablation was less than that of surgical resection (3.9% vs. 46.9%, p<0.001).

Another retrospective study from China included 121 patients, 56 of whom underwent ultrasound-guided percutaneous MWA and 65 who underwent surgical resection (145). Inclusion criteria were: first recurrence after curative hepatectomy, tumors smaller than 5 cm, fewer than 3 tumors, no vascular invasion or extrahepatic disease, Child Pugh A-B, and refusal of liver transplant. Patients with serious medical comorbidities were excluded. Patients received MWA instead of surgical resection if they had an insufficient liver remnant, refused general anesthesia or surgery, or were deemed too high risk for surgical intervention. Interestingly, 40-50% of patients underwent preventative TACE after initial surgery. Median overall survival was similar between the two groups (31.3 months for MWA and 29.4 months for surgery, p=0.405). The incidence of major complications was 13.8% in the surgery group and 5.3% in the MWA group (p<0.001).

In another study, 40 patients with three or fewer metastasis, maximum tumor diameter of 5 cm, and no extrahepatic disease with 64 recurrent lesions were treated with percutaneous RFA (146). Multivariable analysis showed that patients with tumors under 2 cm and recurrence more than one year from curative resection benefited most from therapy. Three patients (7.5%) had a major complication which included two liver abscesses and a biliary stricture. During a median follow-up of 26 months after RFA, 34 (85%) of the patients developed either a local or systemic recurrence. Eight went on to receive repeat RFA, 2 underwent TACE, 6 received systemic chemotherapy, 2 underwent surgery, 5 received chemotherapy and radiation, and 11 received supportive care.

While small and prone to selection bias, these studies did suggest that ablation is safe and can achieve similar survival outcomes to surgical resection in select patients with limited disease. **Table 4** presents a summary of survival outcomes following ablation.

**Table 4:** Survival after ablation for intrahepatic cholangiocarcinoma recurrence in the liver.

Authors	Years	Type	N	Median OS (months)	1-year OS	3-year OS	5-year OS
Chu et al. (146)	1999-2019	Single center	40	26.6	67.2%	36.2%	18.3%
Fu et al. (144)	2000-2011	Single center	12	30	87.5%	37.5%	–
Kim et al. (143)	1999-2009	Single center	20	27.4	70%	–	–
Zhang et al. (140)	2007-2011	Single center	77	21.3	69.8%	20.5%	–

OS, overall survival.

#### Arterially Directed Embolic Therapy

Locoregional intra-arterial therapy is a good option for patients with intrahepatic recurrence who are unable to undergo repeat hepatectomy and may have larger lesions not amenable to ablation. It can also be safely repeated if necessary (147). One retrospective study compared TACE and percutaneous MWA among 275 patients with unresectable intrahepatic recurrence after resection for iCCA (148). Patients were propensity score-matched on tumor markers, tumor size, grade, and extent of resection in addition to other clinical factors. After matching, TACE was associated with better overall (HR=0.69, 95% CI=0.47-0.98) and relapse-free survival (HR=0.69, 95% CI=0.47-0.99). Interestingly, the prognostic factors for TACE and MWA were different. For TACE, tumor size > 5 cm, poor differentiation, and major resection predicted worse survival. For MWA, poor differentiation, infection with hepatitis B, cholelithiasis, and lymph node metastasis were independently associated with survival.

Transarterial radioembolization with labelled ^90^Y can be used for large lesions and for patients with portal vein thrombosis. Sulpice et al. described their single-center experience with ^90^Y (126). The regimen required two stages of arteriography – one to map the hepatic artery, embolize its extrahepatic branches, and quantify hepatopulmonary shunts, and the second to treat the tumor with ^90^Y. The authors noted that treatment with ^90^Y was associated with improved survival after recurrence (p = 0.048). Another study by Mosconi et al. assessed response to ^90^Y in a population of 23 patients with unresectable iCCA, 70% of whom had prior hepatic resections and 34% of whom had prior trans-arterial embolization or chemoembolization (149). After a median follow-up of 16 months, median survival was 17.9 months from date of the radioembolization procedure. Patients who were treatment-naïve did significantly better than patients for whom ^90^Y was preceded by other treatments (52 months vs. 16 months, p=0.009). Of note, 17% of patients received chemotherapy after ^90^Y due to disease progression. In addition, intra-arterial therapy may be useful for patients with multiple recurrences. One study reported that 52.8% of patients who had treatment for recurrent disease and had a second recurrence underwent intra-arterial therapy (10).

#### Stereotactic Body Radiation Therapy (SBRT)

There are currently limited data on the efficacy of SBRT for iCCA and even less evidence in the recurrent setting. Even in the treatment of primary tumors, SBRT is commonly used as a salvage treatment with 29-67% of patients receiving another treatment first (150). It is primarily used as a palliative therapy in patients who are not candidates for other treatments. Jung et al. analyzed outcomes of 30 patients with recurrent cholangiocarcinoma treated with SBRT (151). SBRT doses were 30-60 Gy given in 3-5 fractions. Median survival was 13 months, 1-year overall survival was 53%, and 2-year overall survival was 28%. There was a significant survival difference between patients who recurred within 1 year of surgery (8 months) and patients who recurred more than a year after surgery (17 months) (p=0.007). Time to recurrence was the only significant prognostic variable for overall survival on multivariable analysis. Patients treated with SBRT for recurrent tumors did not have a statistically significant difference in overall survival compared to patients treated for primary tumors, which the authors ascribe to the smaller tumor volume for recurrent tumors. The authors suggest that patients with small and indolent recurrent tumors would most benefit from SBRT as a salvage therapy.

Hypofractionated (proton or photon) external beam radiation therapy also delivers ablative doses. Smart et al. used 37.5-67.5 Gy delivered in 15 fractions in 66 patients with unresectable iCCA, 5 of whom had a prior surgical resection and presented with local recurrence (152). Inclusion criteria included maximum tumor diameter of 12 cm for solitary tumors, no greater than 3 tumors, no extrahepatic disease, and Child-Pugh scores of A and B. Two-year local control was 84%, and 2-year overall survival was 58%. Grade 3+ toxicity was 11%. Only one patient had isolated local failure, but 64% of patients had a disease recurrence. Eight patients were treated with re-irradiation at first recurrence.

#### Combination Therapy

Given anatomic and clinical limitations, combining the above therapies is common, especially resection and ablation. In a study that included 12 centers in the U.S., 47.5% of patients with recurrent iCCA underwent treatment. Among those patients, 75.8% received liver-directed therapy ± systemic chemotherapy. Liver-directed therapy included resection ± ablation (28.5%), only ablation (18.7%), and intra-arterial therapy (52.8%). Patients who underwent repeat resection ± ablation had a median survival of 26.1 months compared to 25.5 months for patients who underwent ablation only and 9.6 months for patients who underwent intra-arterial therapy (p=0.01) (10). Kamphues et al. observed a 51 month overall median survival for 13 patients treated with 12 repeat liver resections and 8 ablations (135).

#### Salvage Liver Transplantation (SLT)

While perihilar cholangiocarcinoma has become an accepted indication for liver transplantation, liver transplantation for iCCA historically has been associated with poor overall survival and recurrence rates as high as 54% (153). However, more recent studies have suggested that favorable outcomes may be achieved in highly selected patients with iCCA, in particular patients with “very early” iCCA measuring ≤ 2 cm who have decompensated cirrhosis and individuals with larger, unresectable iCCA who exhibit stable disease after 6 months of gemcitabine-based chemotherapy. In addition, there are currently several clinical trials currently enrolling patients that are evaluating the role of liver transplantation for patients with iCCA who are not candidates for liver resection, including the exploratory TESLA trial which will include patients with liver-only recurrent iCCA after prior liver resection (154). However, given that there are currently no published data regarding liver transplant for recurrent iCCA, SLT is not a standard treatment modality.

## Conclusion

Among patients with resectable intrahepatic recurrence of CRCLM, HCC, or iCCA, repeat hepatectomy is generally the treatment of choice in selected patients as it has been associated with improved survival versus other treatment modalities. However, among patients with recurrent HCC who cannot tolerate a liver resection due to underlying cirrhosis, salvage liver transplantation provides comparable overall survival and possibly improved recurrence-free survival versus repeat hepatectomy. For patients with tumors ≤ 3 cm in diameter, ablative therapy with RFA or MWA may provide similar survival benefit to repeat hepatectomy and is preferred in patients who are not candidates for major surgery due to comorbidities. While not as efficacious as repeat resection or ablation, TACE, ^90^Y, and SBRT are other liver-directed therapies that can provide improved disease control and a modest survival benefit for patients with recurrent liver disease.

## Author Contributions

CA and ME contributed to the conception and design of the work, acquisition and interpretation of data for the work, drafting and revising of the work, providing approval of the work, and agreeing to be accountable for all aspects of the work in ensuring that questions related to the accuracy or integrity of any part of the work are appropriately investigated and resolved. TP contributed to the conception and design of the work, drafting and revising of the work, providing approval of the work, and agreeing to be accountable for all aspects of the work in ensuring that questions related to the accuracy or integrity of any part of the work are appropriately investigated and resolved. All authors contributed to the article and approved the submitted version.

## Conflict of Interest

The authors declare that the research was conducted in the absence of any commercial or financial relationships that could be construed as a potential conflict of interest.

## Publisher’s Note

All claims expressed in this article are solely those of the authors and do not necessarily represent those of their affiliated organizations, or those of the publisher, the editors and the reviewers. Any product that may be evaluated in this article, or claim that may be made by its manufacturer, is not guaranteed or endorsed by the publisher.
